# Timing of tracheotomy in ICU patients: a systematic review of randomized controlled trials

**DOI:** 10.1186/s13054-015-1138-8

**Published:** 2015-12-04

**Authors:** Koji Hosokawa, Masaji Nishimura, Moritoki Egi, Jean-Louis Vincent

**Affiliations:** Department of Intensive Care, Erasme University Hospital, Université Libre de Bruxelles, Route de Lennik 808, 1070 Brussels, Belgium; Department of Emergency and Critical Care Medicine, Tokushima University Hospital, Tokushima, Japan; Department Intensive Care, Kobe University Hospital, Kobe-city, Hyogo Japan

**Keywords:** Early tracheotomy, Systematic review, Mortality

## Abstract

**Introduction:**

The optimal timing of tracheotomy in critically ill patients remains a topic of debate. We performed a systematic review to clarify the potential benefits of early versus late tracheotomy.

**Methods:**

We searched PubMed and CENTRAL for randomized controlled trials that compared outcomes in patients managed with early and late tracheotomy. A random-effects meta-analysis, combining data from three a priori-defined categories of timing of tracheotomy (within 4 versus after 10 days, within 4 versus after 5 days, within 10 versus after 10 days), was performed to estimate the weighted mean difference (WMD) or odds ratio (OR).

**Results:**

Of the 142 studies identified in the search, 12, including a total of 2,689 patients, met the inclusion criteria. The tracheotomy rate was significantly higher with early than with late tracheotomy (87 % versus 53 %, OR 16.1 (5.7-45.7); p <0.01). Early tracheotomy was associated with more ventilator-free days (WMD 2.12 (0.94, 3.30), p <0.01), a shorter ICU stay (WMD -5.14 (-9.99, -0.28), p = 0.04), a shorter duration of sedation (WMD -5.07 (-10.03, -0.10), p <0.05) and reduced long-term mortality (OR 0.83 (0.69-0.99), p = 0.04) than late tracheotomy.

**Conclusions:**

This updated meta-analysis reveals that early tracheotomy is associated with higher tracheotomy rates and better outcomes, including more ventilator-free days, shorter ICU stays, less sedation, and reduced long-term mortality, compared to late tracheotomy.

**Electronic supplementary material:**

The online version of this article (doi:10.1186/s13054-015-1138-8) contains supplementary material, which is available to authorized users.

## Introduction

Tracheotomy has a number of advantages in patients requiring prolonged mechanical ventilation [1–3], including improved lung mechanics [4, 5], easier oral hygiene, diminished nociceptive stimuli on the larynx or trachea, decreased need for sedatives, enhanced communication, and the fact that the head and neck are free of equipment [6–8]. Tracheotomy, however, also has adverse effects, including procedure-related complications and later cosmetic concerns [9–11].

Because of the relatively complex procedure, tracheotomy was in the past reserved for patients who had been intubated for a long time [12]. However, technological improvements, including simplification and decreased invasiveness of the procedure, have encouraged some to consider a more liberal use of tracheotomy. Some earlier studies in ICU patients suggested that early tracheotomy was associated with better outcomes than late tracheotomy [13–16], but more recent, rigorously designed randomized controlled trials (RCTs) did not show a significant survival benefit [17–19]. The five most recent systematic reviews of RCTs comparing early and late tracheotomy yielded conflicting results [20–24]. However, these meta-analyses combined studies using different timings of early (within 48 hours [16], within 4 days [19], and between 6 and 8 days [17]) versus late interventions, so that the results were difficult to interpret. A meta-analysis in which only studies with early tracheotomy performed within 4 days or 7 days were included reported no significant differences between early and late tracheotomy [24].

Since the most recent systematic reviews were conducted, results from an RCT by Diaz-Prieto et al., which included about 500 patients, have been published [25]. We therefore conducted an updated systematic review and meta-analysis to evaluate the impact of early tracheotomy compared to late tracheotomy on outcome. To investigate whether very early (within 4 days) tracheotomy has a greater impact on outcome than relatively early (within 10 days) tracheotomy, we also evaluated possible differences between very early, relatively early and late tracheotomy.

## Methods

This systematic review was conducted according to the preferred reporting items for systematic reviews and meta-analyses (PRISMA) statement [26]. 

Two authors (KH and ME) searched PubMed and the Cochrane Central Register of Controlled Trials (CENTRAL) on 3 July 2015 using relevant terms (Additional file 1: Table S1). They also independently examined the reference lists from related articles or systematic reviews. Disagreements about eligibility were resolved by consensus. Articles eligible for inclusion were RCTs that compared outcomes associated with early and late tracheotomy. The definition of timing was not specified in the selection step. Studies on pediatric patients, reviews, conference abstracts, quasi-randomized prospective trials and non-English-language articles were excluded. The quality of studies was examined using the method recommended by a Cochrane Collaboration tool for assessing risk of bias in the included RCTs [27].

From the included articles, two of the authors (KH and ME) extracted timing of tracheotomy, number of participating centers, type and number of ICUs, number of patients and the inclusion and exclusion criteria, major disease categories, severity scores, the rate of tracheotomy, the rate of percutaneous dilatation procedures, duration of mechanical ventilation and/or ventilator-free days (VFDs), duration of ICU stay and/or ICU-free days, duration of sedation and/or sedation-free days, the rate of acquired pneumonia, and short-term (defined a priori as ≤2 months) and long-term (defined a priori as >2 months and in hospital) term mortality rates. We also recorded complication rates related to tracheotomy and unplanned extubation. No attempts were made to contact the authors of included studies to obtain missing/unreported data.

Meta-analysis was performed using Review Manager (ver. 5.3, The Nordic Cochrane Center, Copenhagen, Denmark). When continuous values were described by median and IQR or range instead of mean and SD, the following formula was used for approximations:

Mean = Median; SD = IQR/1.35; if 15 < n <70, SD = (b–a)/4, (Minimum (a), Maximum (b)); if n >70, SD = (b–a)/6 [27, 28].

All pooled data were assessed using a random-effects model with an inverse variance method. The estimation of combined continuous values and dichotomous values was described by weighted mean difference (WMD) or odds ratios (OR), respectively, with 95 % CI. We first performed analysis by dividing the data into three groups of studies defined a priori according to the definitions used by the original articles for early versus late timings (within 4 versus after 10 days, within 4 versus after 5 days, or within 10 versus after 10 days) and then combined the results to give an overall estimation of early versus late tracheotomy. Heterogeneity among the included studies was assessed using Tau^2^, Chi^2^ and *I*^2^ tests. A *p* value <0.05 was considered significant.

## Results

Among 142 citations initially identified, 34 studies were selected for full-text reading (see Additional file 1: Fig. S1). Of these, 13 studies were excluded because they were systematic reviews. Nine others were excluded because of unclear inclusion criteria [29], inadequate randomization [30, 31], randomization at different timings and re-allocation to different groups [32], missing patient data before randomization [33, 34], inadequate outcome assessment [35], and non-English-language articles [36, 37]. A total of 12 eligible RCTs [16–19, 25, 38–44] including 2,689 patients were therefore included (Additional file 1: Fig. S1). The studies were similar in terms of quality assessment (Additional file 1: Fig. S2).

The definitions of early and late tracheotomy varied among the studies (Table 1). Seven studies used very early tracheotomy (within 4 days) [16, 19, 38, 40–43] and five used early tracheotomy (within 10 days) [17, 18, 25, 39, 44]. Late tracheotomy was defined as after 10 days in 10 RCTs [16–19, 25, 38–41, 44] and as after 5 days in 2 studies [42, 43]. The studies included different patient populations, including patients with intracranial disease [43], trauma [39], burns [38], and postoperative patients [18, 41, 42] (Table 1). Some studies excluded patients with pneumonia [17, 41, 42, 44]. Tracheotomy was performed primarily using percutaneous methods in 9 of the 11 studies [16–19, 25, 41–44] that provided this information (Table 1). The reported incidence of complications related to tracheotomy ranged from 0 % to 39 %, with the most frequent reported complication being bleeding (data not shown).

**Table 1:** Summary of the included randomized controlled trials of early versus late tracheotomy

Study						Patients			
	Definition of early versus late tracheotomy^a^(days)	Type of ICU; number of ICUs	Number of patients, early versus late groups	Inclusion criteria	Excluded	Major disease category	APACHE II/SAPS II	Tracheotomy rate (number (%)) in early versus late groups	Percutaneous dilatation tracheotomy (number (%)) in early versus late groups
Saffle et al. (2002) [38]	2-4 vs. 14–16	Burn; 1	21 vs. 23	High predicted probability of prolonged MV		Burn (100 %)	NA	21 (100 %) vs. 16 (70 %)	NA
Rumbak et al. (2004) [16]	≤2 vs. >14	Medical; 2	60 vs. 60	exp. >14 d MV; APACHE II >25		Respiratory failure (100 %), severe sepsis (68 %)	26.9	60 (100 %) vs. 50 (83 %)	All in both groups
Barquist et al. (2006) [39]	<8 vs. >28	Trauma; 1	29 vs. 31	GCS >4 with no head injury; GCS >9 with head injury		Trauma (100 %)	12.6	27 (93 %) vs. 11 (35 %)	0/27 (0 %) vs. 0/11 (0 %)
Blot et al. (2008) [40]	≤4 vs. >14	Medical and surgical; 25	61 vs. 62	exp. >7 d MV	Irreversible neurological disease	Respiratory failure (33 %), neurology (23 %), trauma (19 %)	NA/50	60 (98 %) vs. 16 (26 %)	19/60 (32 %) vs. 7/16 (44 %)
Terragni et al. (2010) [17]	6-8 vs. 13–15	NA; 12	209 vs. 210	SAPS II = 35–65; SOFA ≥5; worsening respiratory conditions; unchanged/worse SOFA sore	Pneumonia (CPIS ≥6); COPD	Respiratory failure (46 %), neurology (24 %), cardiovascular disease (23 %)	NA/50.4	145 (69 %) vs. 119 (57 %)	141/145 (97 %) vs. 113/119 (95 %)
Trouillet et al. (2011) [18]	<5-7 vs. >19	Surgical; 1	109 vs. 107	exp. >7 d MV	Irreversible neurologic disorder	Post-cardiac surgery (100 %)	NA/46.5	109 (100 %) vs. 29 (27 %)	All in both groups
Zheng et al. (2012) [41]	3 vs. 15	Surgical; 1	58 vs. 61	PaO_2_/FiO_2_ <200; APACHE II >15;SOFA >5; CPIS >6; exp. >14 d MV	Pulmonary infection (CPIS >6)	NA	20.0	58 (100 %) vs. 51 (84 %)	All in both groups
Koch et al. (2012) [42]	≤4 vs. ≥6	Surgical; 1	50 vs. 50	exp. >21 d MV	Pneumonia	Neurosurgical (28 %), trauma (25 %)	22	All in both groups	All in both groups
Young et al. (2013) [19]	≤4 vs. >10	General;70 and surgical; 2	451 vs. 448	exp. >7 d MV	Respiratory failure due to chronic neurological disease	Pulmonary (60 %), gastrointestinal (19 %)	19.8	418 (93 %) vs. 204 (46 %)	378/418 (90 %) vs. 176/204 (86 %)
Bösel et al. (2013) [43]	≤3 vs. 7–14	Neuro; 1	30 vs. 30	ICH; SAH; or AIS; exp. >14 d MV	Severe chronic cardiopulmonary disease; extensive brainstem lesions	Non-traumatic neurology (100 %)	17	30 (100 %) vs. 18 (60 %)	27/30 (90 %) vs. 16/18 (89 %)
Mohamed et al. (2014) [44]	≤10 vs. >10	NA; 2	20 vs. 20	APACHE ≥15	Pneumonia	TBI (43 %), CVA (25 %)	24	All in both groups	All in both groups
Diaz-Prieto et al. (2014) [25]	<8 vs. >14	NA; 4	245 vs. 244	1, exp. >7 d MV; 2, attending physician’s acceptance at 3–5 d		Respiratory insufficiency (60 %), coma (22 %)	20	167 (68 %) vs. 135 (55 %)	All in both groups

^a^Values are shown as days from the initiation of mechanical ventilation, except one that used days from ICU admission [19]. *AIS* acute ischemic stroke, *APACHE* acute physiology and chronic health evaluation, *COPD* chronic obstructive pulmonary disease, *CPIS* clinical pulmonary infection score, *CVA* cerebrovascular accident, *d* days, *exp*. expected, *GCS* Glasgow coma scale, *ICH* intracerebral hemorrhage, *MV* mechanical ventilation, *NA* not available, *PaO*_*2*_ */FiO*_*2*_ partial pressure arterial oxygen/fraction of inspired oxygen, *RCT* randomized controlled trial, *SAH* subarachnoid hemorrhage, *SAPS* simplified acute physiology score, *SOFA* sequential organ failure assessment

### Meta-analysis results

#### Tracheotomy rate

**Fig. 1:**
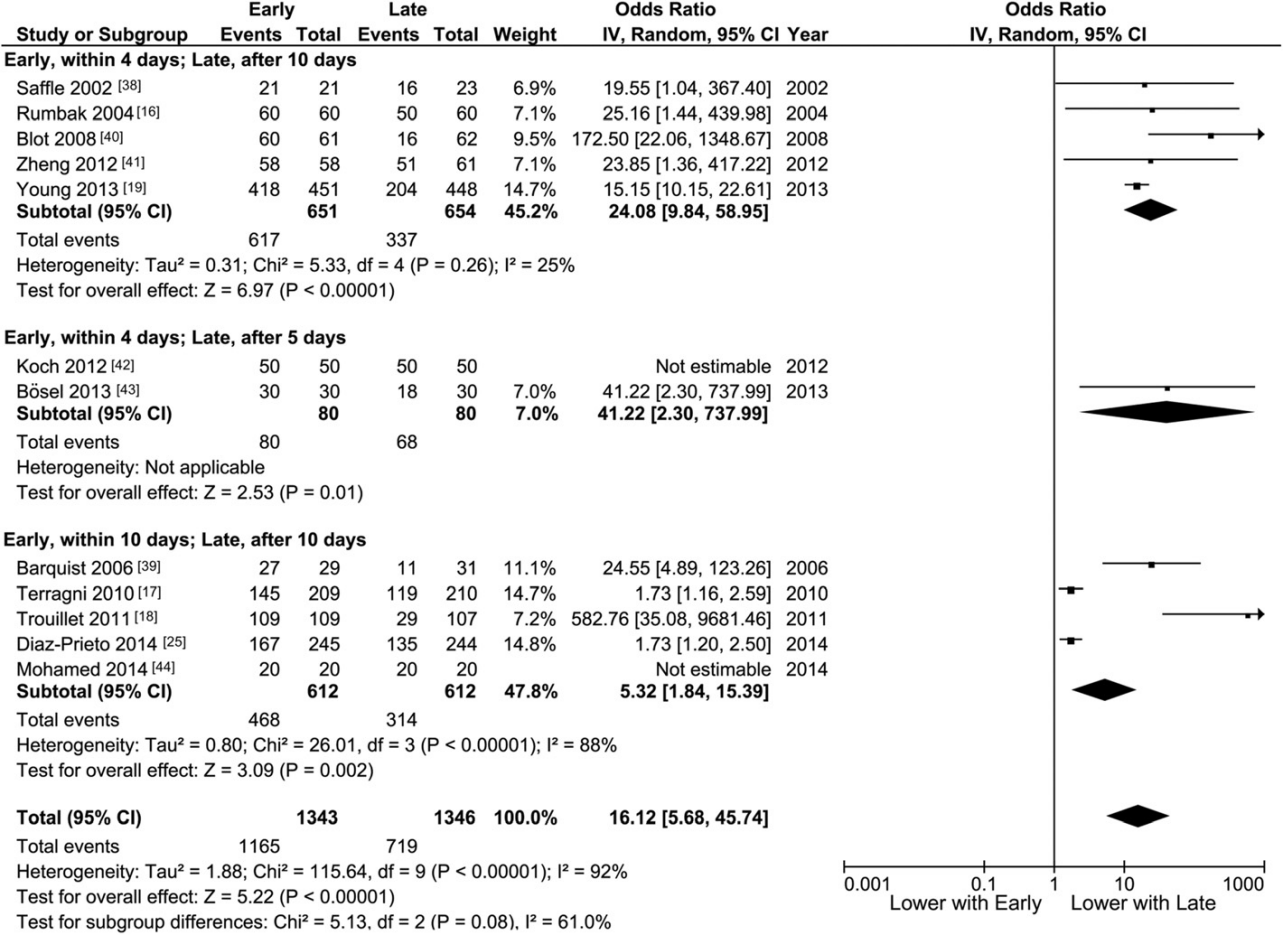
Tracheostomy rate. Meta-analysis of the 12 studies. *I-V* inverse variance

The rate of tracheotomy was significantly higher with early than with late tracheotomy in studies comparing timings of within 4 versus after 10 days (95 % versus 52 %, OR 24.08) and in those comparing within 10 versus after 10 days (76 % versus 51 %, OR 5.32, Fig. 1). When the data were combined for the 12 studies [16–19, 25, 38–44], the rates were 87 % for early versus 53 % for late tracheotomy (OR 16.12 (5.68, 45.74), *p* <0.01; I^2^ 92 %, *p* heterogeneity <0.01).

#### Mechanical ventilation

**Fig. 2:**
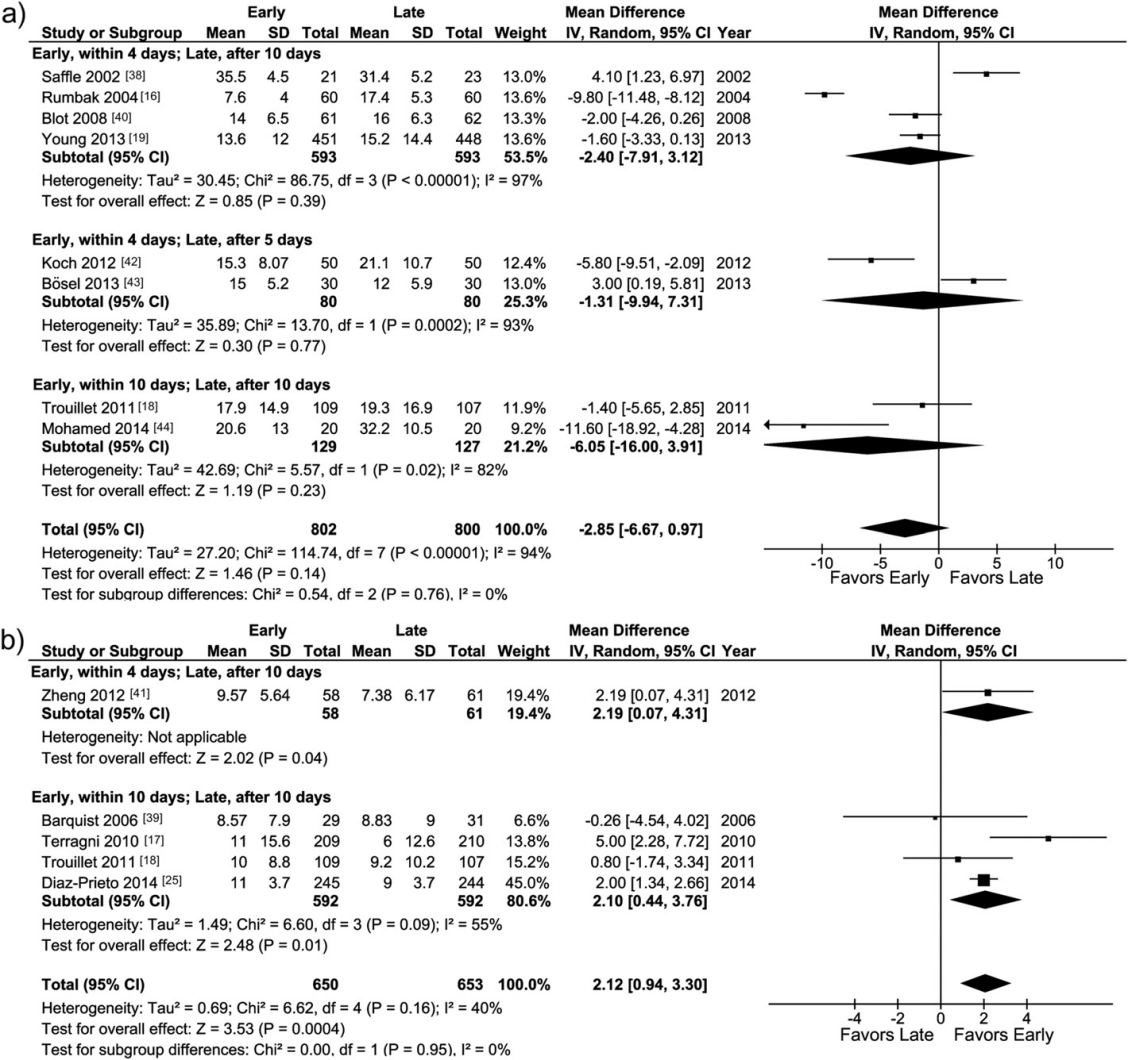
**a** Duration of mechanical ventilation. Meta-analysis of the eight studies providing this information. **b** Ventilator-free days. Meta-analysis of the five studies providing this information. *I-V* inverse variance

The duration of mechanical ventilation was reported in eight studies [16, 18, 19, 38, 40, 42–44] (Table 2) and did not differ significantly between the early and late tracheotomy groups in any of the three predefined groups of studies or overall (Fig. 2a). VFDs were reported in five studies [17, 18, 25, 39, 41] (Table 2) and were greater with early than with late tracheotomy in one of the predefined groups of studies (within 10 versus after 10 days; WMD 2.10 (0.44, 3.76), *p* <0.01; *I*^2^ 55 %, *p* heterogeneity = 0.09; Fig. 2b) and overall (WMD 2.12 (0.94, 3.30), *p* <0.01; *I*^2^ 40 %, *p* heterogeneity = 0.16; Fig. 2b).

**Table 2:** Reported outcomes in the included randomized controlled trials

	Duration of mechanical ventilation, early versus late groups (days)	Number of ventilator-free days in 28 days, early versus late groups	Duration of ICU stay, early versus late groups (days)	Number of ICU-free days in 28 days, early versus late groups	Duration of sedation, early versus late groups (days)	Number of sedation-free days in 28 days, early versus late groups	Acquired pneumonia, early versus late groups	Mortality (≤2 months), early versus late groups	Mortality (>2 months), early versus late groups	Other outcomes, early versus late groups,
Saffle et al. (2002) [38]	35.5 (4.5) vs. 31.4 (5.2) (*p*, NA)	NA	NA	NA	NA	NA	21 (100 %) vs. 22 (96 %) (*p* = 0.16)	17 (81 %) vs. 17 (74 %) (*p* = 0.58)	NA	Successfully extubated, 1 (5 %) vs. 6 (26 %) (*p* <0.01)
Rumbak et al. (2004) [16]	7.6 (4.0) vs. 17.4 (5.3) (*p* <0.01)	NA	4.8 (1.4) vs. 16.2 (3.8) (*p* <0.01)	NA	3.2 (0.4) vs. 14.1 (2.9) (*p* <0.01)	NA	3 (5 %) vs. 15 (25 %) (*p* <0.05)	19 (32 %) vs, 37 (62 %) (*p* <0.05) (at 30 d)	NA	Damage to the larynx and lips, rated 0–1 vs. 2–3
Barquist et al. (2006) [39]	NA	8.57 (7.9) vs. 8.83 (9) (in 30 d) (*p* = 0.9)	NA	5.0 (6.0) vs. 5.3 (6.5) (in 30 d) (*p* = 0.8)	NA	NA	28 (97 %) vs. 28 (90 %) (*p* = 0.6)	2 (6.9 %) vs. 5 (16 %) (p = 0.4)	NA	
Blot et al. (2008) [40]	14 (2–28) vs. 16 (3–28) (*p* = 0.62)	NA	NA	NA	NA	18 (0–27) vs. 15 (0–27)	30 (49 %) vs. 31 (50 %) (*p* = 0.94)	12 (20 %) vs. 15 (24 %) (at 28 d); 16 (27 %) vs. 15 (24 %) (at 60 d)	NA	Laryngeal symptoms, 1 (2 %) vs. 7 (11 %) (*p* = 0.01)
Terragni et al. (2010) [17]	NA	11 (0–21) vs. 6 (0–17) (*p* = 0.02)	NA	0 (0–13) vs. 0 (0-8) (*p* = 0.02)	NA	NA	30 (14 %) vs. 44 (21 %) (*p* = 0.07)	55 (26 %) vs. 66 (31 %) (*p* = 0.25) (at 28 d)	72/144 (50 %) vs. 75/138 (57 %)(*p* = 0.25) (in 1 year)	Successful weaning, 161 (77 %) vs. 142 (68) *(p* = 0.02)
Trouillet et al. (2011) [18]	17.9 (14.9) vs. 19.3 (16.9) (*p* = 0.55)	10.0 (8.8) vs. 9.2 (10.2) (*p* = 0.52)	23.9 (21.3) vs. 25.5 (22.2) (*p* = 0.85)	NA	6.4 (5.9) vs. 9.6 (7.3) (*p* <0.01)	19.0 (9.1) vs. 15.5 (9.3) (*p* <0.01)	50 (46 %) vs. 47 (44 %) (*p* = 0.77)	17 (16 %) vs. 23 (21 %) (*p* = 0.30) (at 30 d)	12/74 (16 %) vs. 17/74 (23 %) (*p* = 0.49) (in 2.4 years in mean)	ADL, anxiety, depression, or PTSD, similar
Zheng et al. (2012) [41]	NA	9.6 (5.6) vs. 7.4 (6.2) (p = 0.05)	NA	8.0 (5.0–12.0) vs. 3.0 (0–12.0) (*p* <0.01)	NA	20.8 (2.4) vs. 17.1 (2.3) (*p* = 0.05)	17 (29 %) vs. 30 (49 %) (*p* = 0.03)	8 (14 %) vs. 6 (10 %) (*p* = 0.55) (at 28 d)	NA	
Koch et al. (2012) [42]	15.3 (9.1–19.8) vs. 21.1 (13.5–27.9) (*p* ≤0.01)	NA	21.5 (15.0–30.0) vs. 30.6 (22.0–37.0) (*p* ≤0.05)	NA	NA	NA	19 (38 %) vs. 32 (64 %)	9 (18 %) vs. 7 (14 %) (*p* = 0.79) (in ICU)	10 (20 %) vs. 11(22 %) (*p* = 0.81) (in hospital)	
Young et al. (2013) [19]	13.6 (12.0) vs. 15.2 (14.4) (*p* = 0.06)	NA	13.0 (8.2–19.1) vs. 13.1 (7.4–23.6) (*p* = 0.74) in survivors; 9.3 (4.2–16.0) vs. 10.4 (6.0–19.7) (*p* = 0.16) in non-survivors	NA	5 (3–9) vs. 8 (4–12) (*p* <0.01) in survivors; 5 (3–9) vs. 6 (4–10) (*p* = 0.11) in non-survivors	NA	NA	139 (31 %) vs. 141 (32 %) (*p* = 0.89) (at 30 d)	168 (40 %) vs. 180 (41 %) (*p* = 0.63) (in hospital); 207 (46 %) vs. 217 (49 %) (*p* = 0.38) (1 year)	Antibiotic use, 5 (1–8) vs. 5 (1–10) (*p* = 0.95) (in 30 d)
Bösel et al. (2013) [43]	15 (10–17) vs. 12 (8–16) (*p* = 0.23)	NA	17 (13–22) vs. 18 (16–28) (*p* = 0.38)	NA	NA	NA	NA	3 (10 %) vs. 14 (47 %) (*p* <0.01) (in ICU)	8 (27 %) vs. 18 (0.6 %) (*p* = 0.02) (in 6 months)	Sedation use (42 %) vs. (62 %) (*p* = 0.02).
Mohamed et al. (2014) [44]	20.6 (13.0) vs. 32.2 (10.5) (*p* <0.01)	NA	21.1 (13.5) vs. 40.2 (12.7) (*p* <0.01)	NA	NA	NA	4 (20 %) vs. 8 (40 %)	NA	8 (40 %) vs. 8 (40 %) (in hospital)	
Diaz-Prieto et al. (2014) [25]	NA	11 (0–22) vs. 9 (0–22) (*p* = 0.05)	22 (6–101) 22.5 (6–174) (*p* = 0.31)	NA	11 (2–92) vs. 14 (0–79) (*p* = 0.02)	NA	33 (13 %) vs. 23 (9 %) (*p* = 0.16)	42 (17 %) vs. 47 (19 %) (*p* = 0.54) (at 28 d)	63 (26 %) vs. 73 (30 %) (*p* = 0.30) (at 90 d); 67 (27 %) vs. 78 (32 %) (*p* = 0.26) (in hospital)	Excluded by attending physician, 284 (58 %)

The values are presented as number (%), mean with (SD) or median with (IQR). The values indicate early tracheostomy versus late tracheostomy

*ADL* activities of daily living, *d* days, *NA* not available, *PTSD* posttraumatic stress disorder, *RCT* randomized controlled trial

#### ICU stay

The duration of ICU stay was reported in seven studies [16, 18, 19, 25, 42–44] (Table 2) and was significantly shorter with early than with late tracheotomy overall (WMD –5.14 (–9.99, –0.28), *p* = 0.04; *I*^2^ 96 %, *p* heterogeneity <0.01; Additional file 1: Figure S3a). Three studies reported ICU-free days [17, 39, 41]: there were no significant differences with early compared to late tracheotomy overall (Additional file 1: Figure S3b).

#### Sedation

**Fig. 3:**
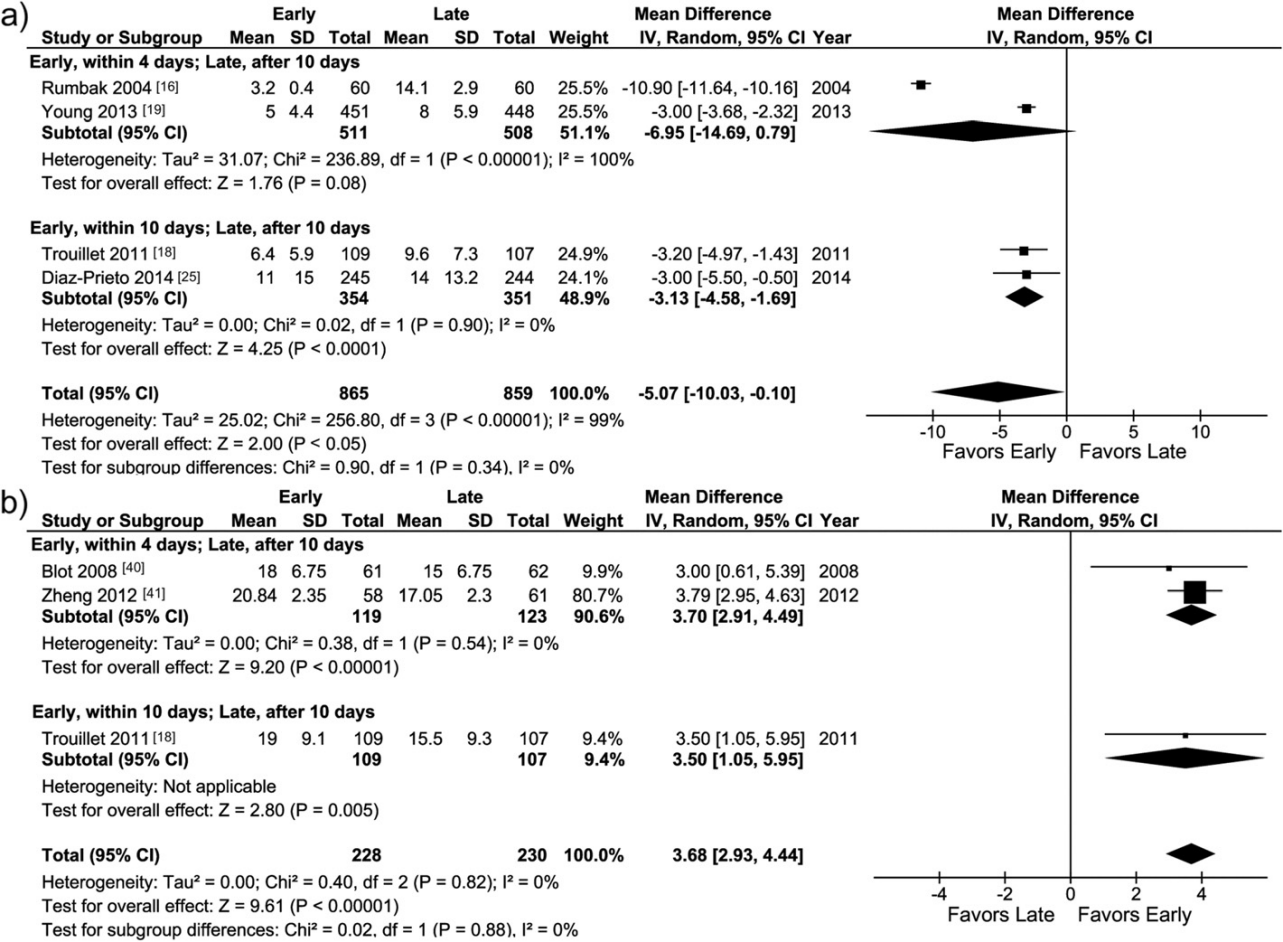
**a** Duration of sedation. Meta-analysis of the four studies providing this information. **b** Sedation-free days. Meta-analysis of the three studies providing this information. *I-V* inverse variance

The duration of sedation was reported in four studies [16, 18, 19, 25] (Table 2) and was shorter with early than with late tracheotomy in one of the predefined groups of studies (within 10 versus after 10 days) and overall (WMD –5.07 (–10.03, –0.10), *p* <0.05; *I*^2^ 99 %, *p* heterogeneity <0.01; Fig. 3a). The number of sedation-free days was reported in three studies [18, 40, 41] and was larger with early than with late tracheotomy in two of the predefined groups of studies (within 4 versus after 10 days, and within 10 versus after 10 days) and overall (WMD 3.68 (2.93, 4.44), *p* <0.01; *I*^2^ 0 %, *p* heterogeneity = 0.82; Fig. 3b).

#### Acquired pneumonia

The risk of acquired pneumonia was reported in 10 studies [16–18, 25, 38–42, 44] (Table 2) and did not differ in any of the predefined groups of studies, or overall (OR 0.69 (0.45, 1.06), *p* = 0.09; *I*^2^ 60 %, *p* heterogeneity <0.01; Additional file 1: Figure S4).

#### Mortality

**Fig. 4:**
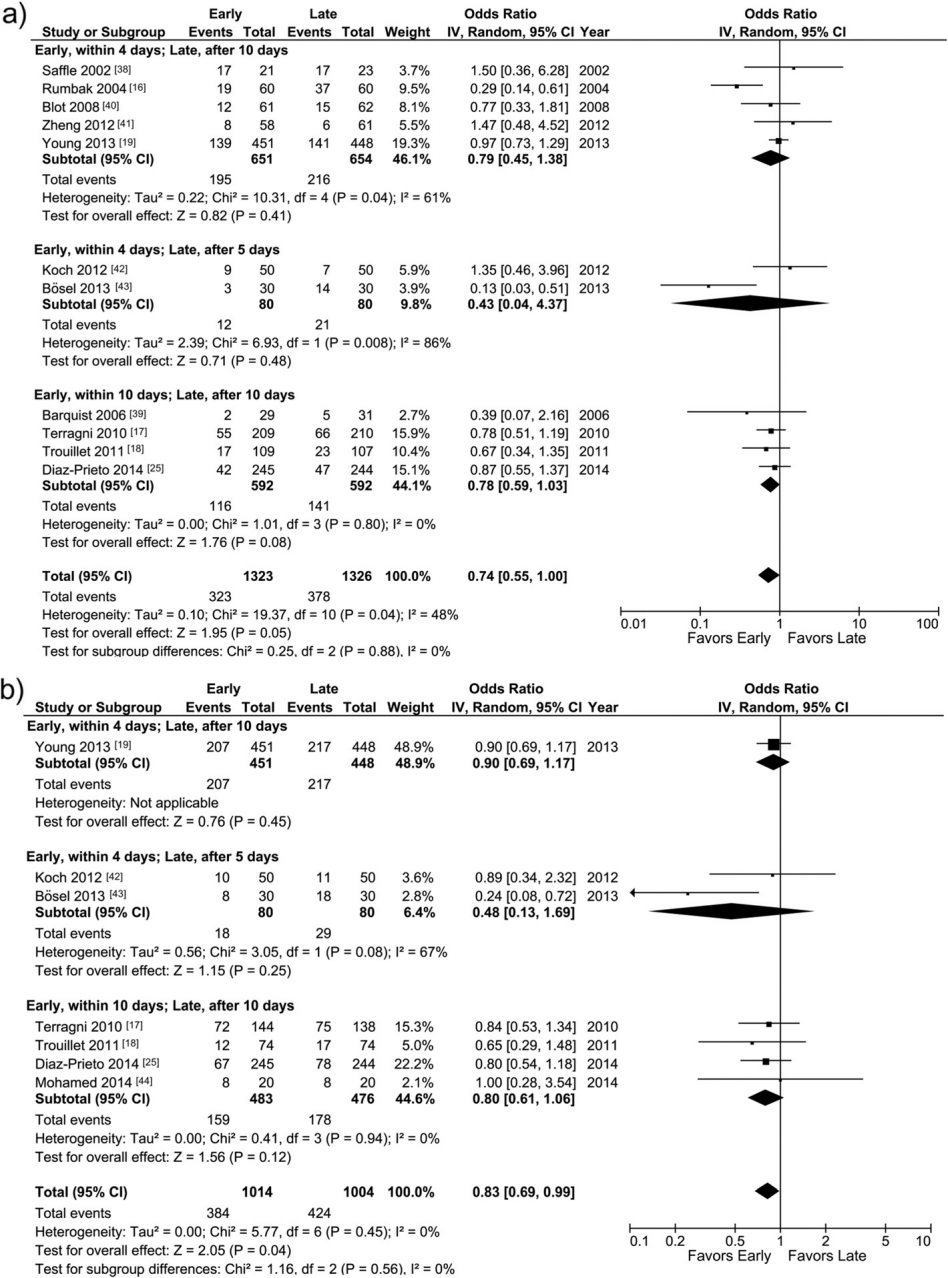
**a** Short-term mortality. Meta-analysis of the 11 studies providing this information. **b** Long-term mortality. Meta-analysis of the 7 studies providing this information. Data for 28-day, 30-day and ICU mortality were combined to show short-term mortality, and data for 1-year, 6-month and hospital mortality were combined as long-term mortality. *I-V* inverse variance

Short-term (≤2 months) mortality rates were reported in 11 studies [16–19, 25, 38–43] (Table 2) and did not differ in any of the predefined groups of studies or overall (OR 0.74 (0.55, 1.00), *p* = 0.05; *I*^2^ 48 %, *p* heterogeneity = 0.04; Fig. 4a). Long-term (>2 months) mortality rates were reported in seven studies [17–19, 25, 42–44] and did not differ in any of the predefined groups of studies but were significantly lower with early than with late tracheotomy overall (OR 0.83 (0.69, 0.99), *p* = 0.04; *I*^2^ 0 %, *p* heterogeneity = 0.45; Fig. 4b).

## Discussion

Our analysis indicated that early (versus late) tracheotomy was associated with a larger number of VFDs, shorter ICU stay, shorter duration of sedation and lower long-term mortality rates.

Our meta-analysis included a recently published study [25] and gathered a larger number of patients than other recent systematic reviews [20–24], thus improving the ability of the analysis to show differences in major outcomes. Pooled outcome data from most of these reviews did not show a significant reduction in mortality [20, 21, 23, 24], rates of pneumonia [20–24] or duration of mechanical ventilation [20–24] with early compared to late tracheotomy, but one meta-analysis did show significantly reduced long-term mortality [22]. The inclusion of the large study by Diaz-Prieto et al. [25], which included about 500 ICU patients, enabled us to highlight some interesting new differences in outcomes between these two groups of patients. We used robust statistical analysis, including a random-effects model in which the weights of small and large studies are taken into account.

The definition of early and late in previous systematic reviews was before versus after 1 week [23] or 10 days [20–22, 24]. Our broader definitions enabled us to include the study by Koch et al., in which very early (within 4 days) tracheotomy was compared to relatively early (after 5 days) tracheotomy [42]. We also included the study by Bösel et al., who compared very early tracheotomy (within 1–3 days after intubation) to what the authors called "standard" timing (between days 7 and 14) [43]. These studies would have been excluded if we had limited the late tracheotomy group to more than 7 or 10 days. Moreover, our cutoffs for the timing of tracheotomy produced some interesting findings in the differences between very early and moderately early procedures.

Tracheotomy rates were generally lower in the late tracheotomy than in the early tracheotomy groups, likely because patients will have recovered or died by the later time point. In addition, there is no reliable means of predicting the likely length of mechanical ventilation. The differences in tracheotomy rates between the early and late group were much larger in the predefined group of studies comparing within 4 days versus after 10 days than that comparing within 10 versus after 10 days.

Our results showed that early tracheotomy was associated with a larger number of VFDs in the group of studies comparing tracheotomy within 10 versus after 10 days. This seems to contradict the policy that tracheotomy should be delayed until after 14 days [7], but does support several reviews that suggest that the need for tracheotomy should be assessed on a daily basis with a definite decision being taken as early as 4–7 days after endotracheal intubation [9, 45, 46].

As in previous meta-analyses [20, 21], early tracheotomy was associated with a shorter duration of sedation. Some [47–49], but not all [50], retrospective observational studies have also reported that early tracheotomy allows a shorter duration of sedation. These differences may be related to the sedation strategies used in these studies.

Our analysis has several limitations. First, there was marked heterogeneity among studies for some of the outcome measures, likely related to the diverse patient groups and characteristics and the different timings of tracheotomy, which are inherent in all systematic reviews on this topic, and the fact that respiratory management may have changed between 2002 and 2015, the dates of publication of the included studies. Second, early tracheotomy may be particularly beneficial in selected groups of patients, such as those with head or spinal cord injury or massive stroke [6, 51], but our meta-analysis could not address this question. Third, adverse effects and cost-effectiveness were not assessed. Finally, the statistical plan included the estimation of WMD using approximate SD values calculated from the IQR.

## Conclusions

This updated meta-analysis reveals that early tracheotomy is associated with a significantly higher rate of tracheotomy and a larger number of VFDs, shorter ICU stays, shorter duration of sedation and lower long-term mortality rates than late tracheotomy. The assessment restricted to groups of studies with different time cutoffs did not provide enough information to be able to draw conclusions about differences between very early (within 4 days) and moderately early (within 10 days) tracheotomy.

## Key messages

- Early tracheotomy was associated with significantly higher rates of tracheotomy than late tracheotomy
- Early tracheotomy is associated with a larger number of VFDs, shorter ICU stays, shorter duration of sedation and lower long-term mortality rates than late tracheotomy
- In the group of studies that compared tracheotomy within 10 versus after 10 days, early tracheotomy was associated with more VFDs than late tracheotomy

## Additional file

Additional file 1: Table S1. Electronic database search strategy and results. Fig. S1 Preferred reporting items for systematic reviews and meta-analyses (PRISMA) study flow chart. Fig. S2 The quality assessment of included studies. Fig. S3 **a** Duration of ICU stay. Meta-analysis of the seven studies providing this information. **b** ICU-free days. Meta-analysis of the three studies providing this information. CI confidence interval, *I-V* inverse variance, *SD* standard deviation. Fig. S4 The incidence of acquired pneumonia. Meta-analysis of the 10 studies providing this information. (DOCX 1069 kb)

## Abbreviations

ADL: activities of daily living
AIS: acute ischemic stroke
APACHE: acute physiology and chronic health evaluation
CENTRAL: Cochrane Central Register of Controlled Trials
CI: confidence interval
COPD: chronic obstructive pulmonary disease
CPIS: clinical pulmonary infection score
CVA: cerebrovascular accident
GCS: Glasgow coma scale
ICH: intracerebral hemorrhage
ICU: intensive care unit
IQR: interquartile range
MV: mechanical ventilation
OR: odds ratio
PaO_2_/FiO_2_: partial pressure arterial oxygen/fraction of inspired oxygen
PRISMA: preferred reporting items for systematic reviews and meta-analyses
PTSD: posttraumatic stress disorder
RCT: randomized controlled trial
SAH: subarachnoid hemorrhage
SAPS: simplified acute physiology score
SD: standard deviation
SOFA: sequential organ failure assessment
VFD: ventilator-free day
WMD: weighted mean difference
