# Supplementary material for: Timing of tracheotomy in ICU patients: a systematic review of randomized controlled trials
Source: Crit Care. 2015 Dec 4;19:424. doi: 10.1186/s13054-015-1138-8 (PMC4669624; doi:10.1186/s13054-015-1138-8)

**Additional file**

**Timing of tracheotomy in ICU patients:**

**a systematic review of randomized controlled trials**

Koji Hosokawa, Masaji Nishimura, Moritoki Egi, Jean-Louis Vincent

Table S1. Electronic database search strategy and results

|  |  | Terms | Citations (n) |
| --- | --- | --- | --- |
| A) PubMed, searched on July 3rd, 2015. | | | |
|  | #1 | early tracheostomy | 1228 |
|  | #2 | early tracheotomy | 636 |
|  | #3 | #1 OR #2 | 1705674 |
|  | #4 | random* | 974300 |
|  | #5 | #3 AND #4 | 102 |
|  |  |  |  |
| B) Cochrane Central Register of Controlled Trials, searched on July 3rd, 2015. | | | |
|  | #1 | early tracheostomy or early tracheotomy in Trials | 76 |

Figure S1. PRISMA study flow chart.

PubMed, 102

CENTRAL, 76

Additional records identified in references, 6

Records after duplicates removed, 142

Full-text assessed, 34

Not eligible from title and abstract, 108

Included studies (compared early versus late), 12

# within 4 versus after 10 days (5 trials; n=1,305)

# within 4 versus after 5 days (2 trials; n=160)

# within 10 versus after 10 days (5 trials; n=1,224)

Systematic review, 13

Unclear inclusion criteria, 1

Quasi-randomized, 3

Inadequate participant follow-up, 2

Inadequate result assessment, 1

Non-English, 2

Figure S2. The quality assessment of included studies


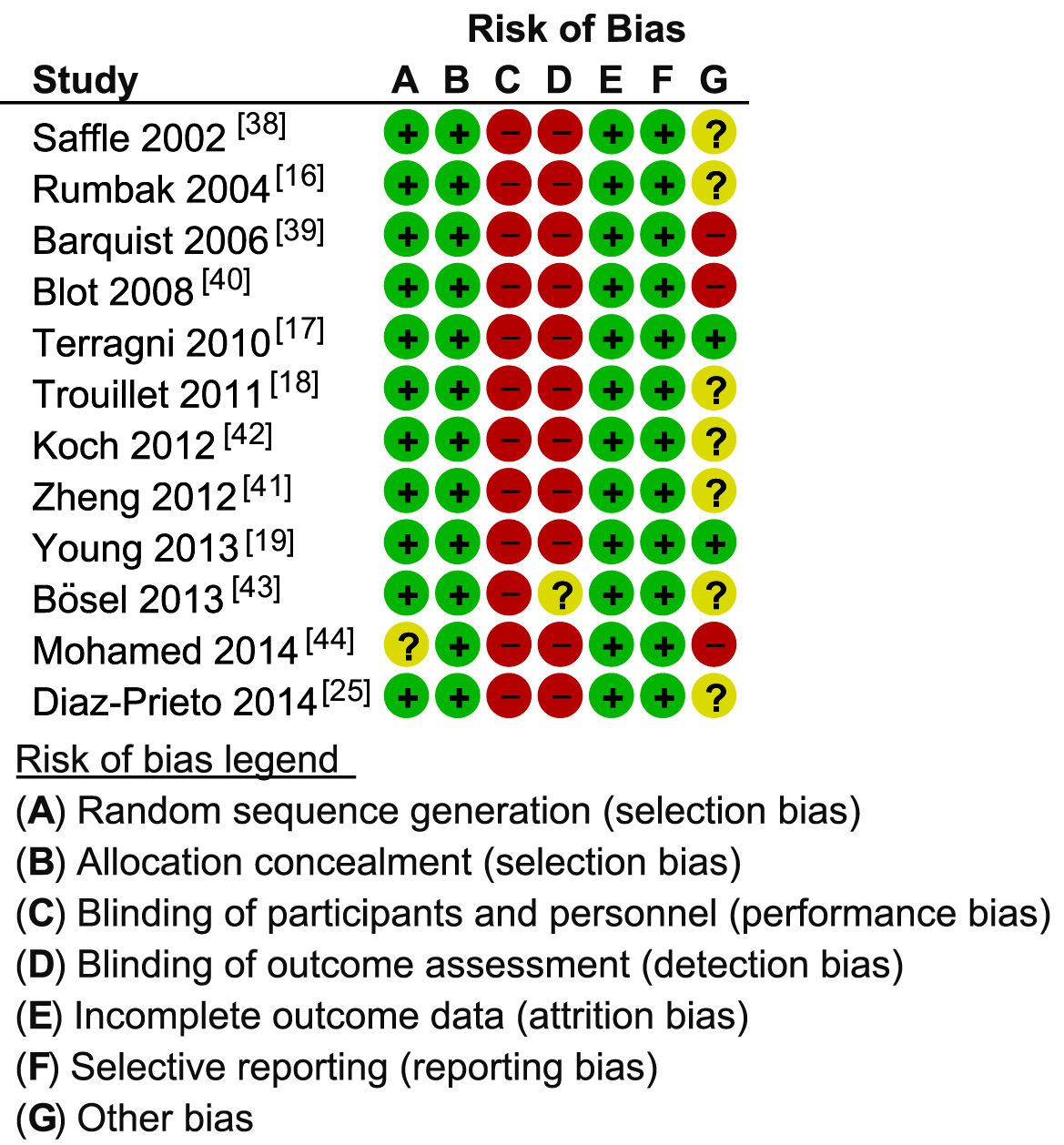


+, low risk of bias; ?, unclear risk of bias; –, high risk of bias.

Figure S3.

a. Duration of ICU stay. Meta-analysis of the 7 studies providing this information.

b. ICU-free days. Meta-analysis of the 3 studies providing this information.

CI, confidence interval; I-V, inverse variance; SD, standard deviation.


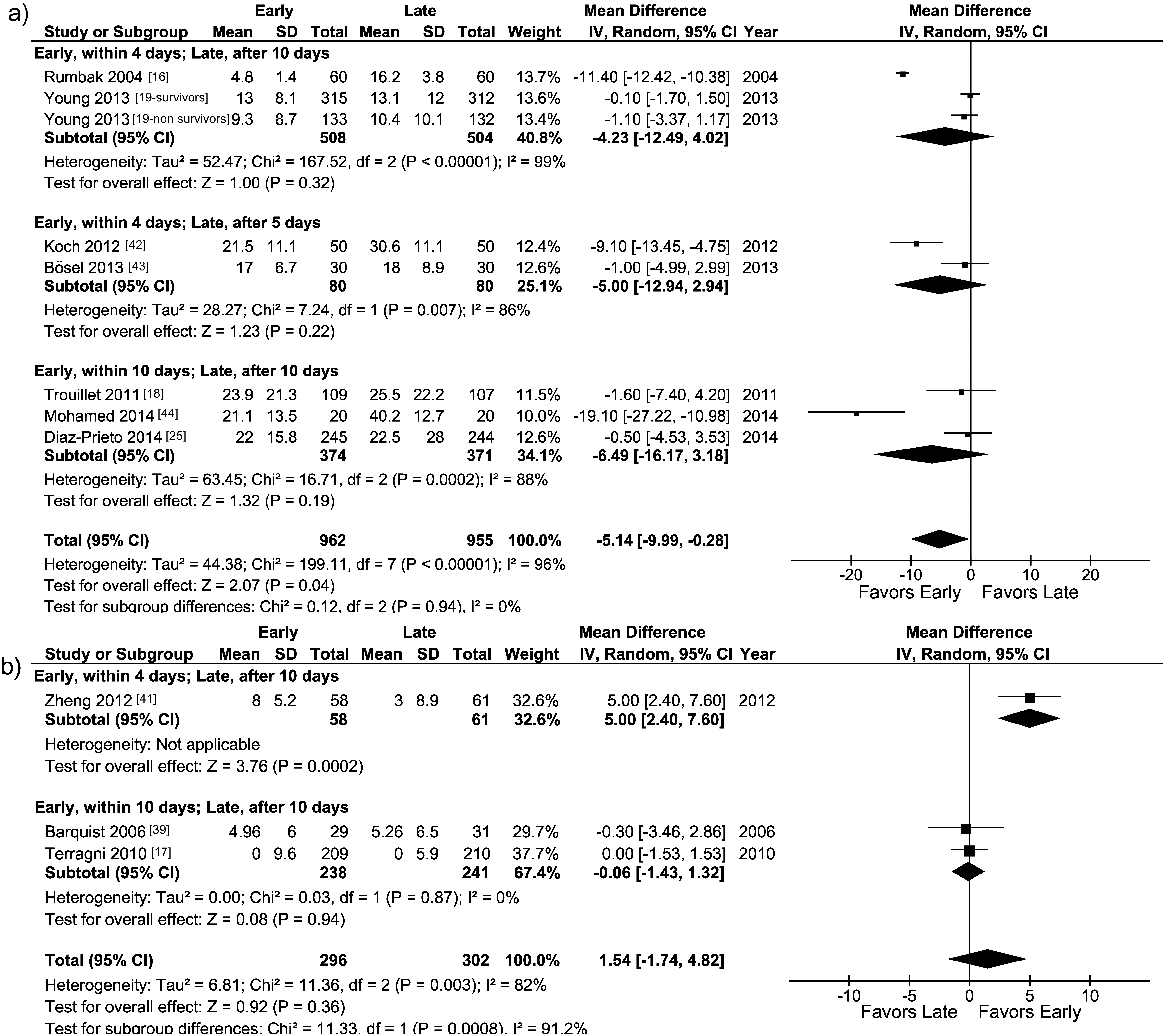


Figure S4. The incidence of acquired pneumonia. Meta-analysis of the 10 studies providing this information.

CI, confidence interval; I-V, inverse variance.


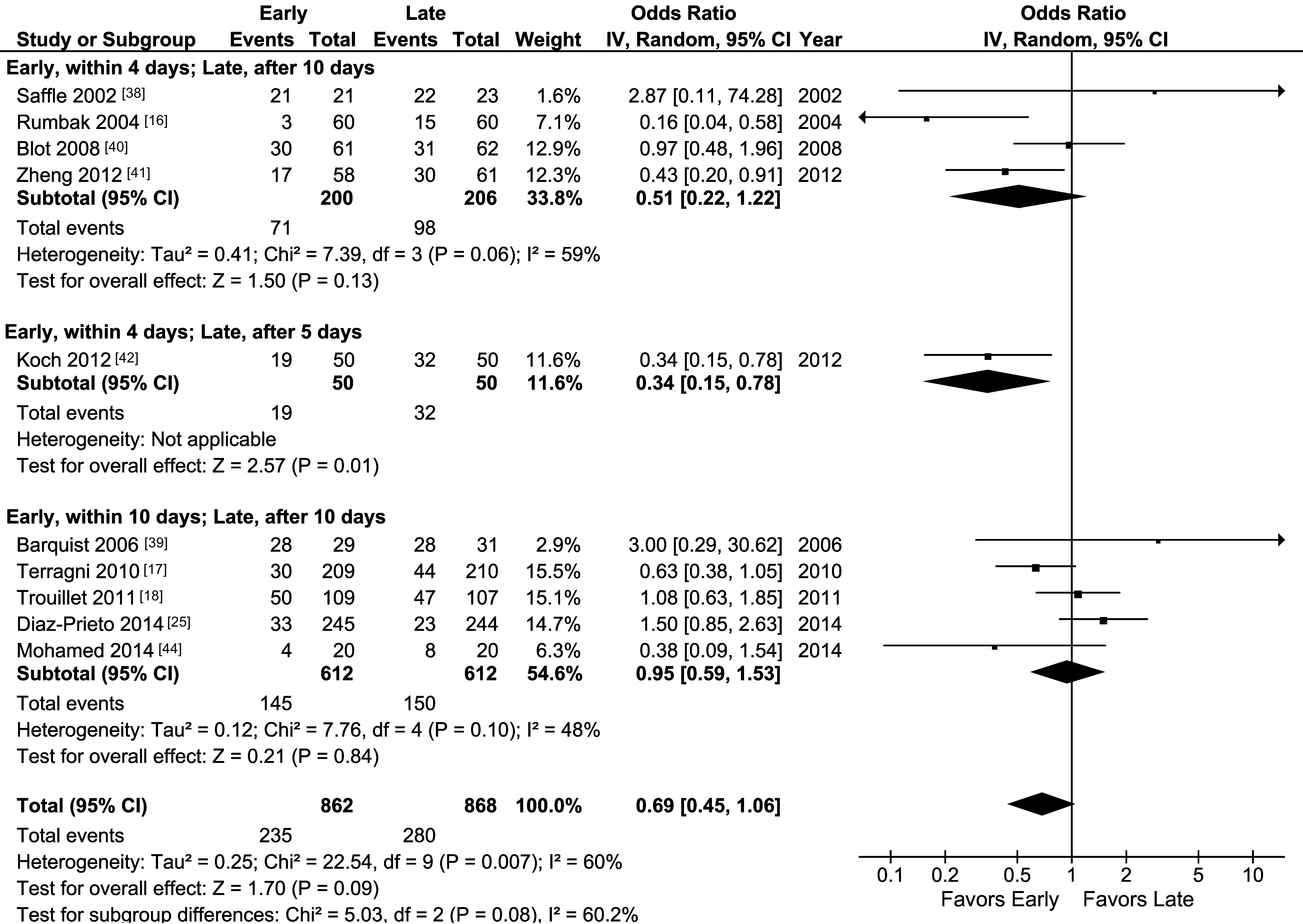

Supplement: Additional file 1: Table S1. — Electronic database search strategy and results. Fig. S1 Preferred reporting items for systematic reviews and meta-analyses (PRISMA) study flow chart. Fig. S2 The quality assessment of included studies. Fig. S3 a Duration of ICU stay. Meta-analysis of the seven studies providing this information. b ICU-free days. Meta-analysis of the three studies providing this information. CI confidence interval, I-V inverse variance, SD standard deviation. Fig. S4 The incidence of acquired pneumonia. Meta-analysis of the 10 studies providing this information. (DOCX 1069 kb) [file 13054_2015_1138_MOESM1_ESM.docx]
